# Supplementary material for: Teaching CORnet human fMRI representations for enhanced model-brain alignment
Source: Cogn Neurodyn. 2025 Apr 15;19(1):61. doi: 10.1007/s11571-025-10252-y (PMC11999921; doi:10.1007/s11571-025-10252-y)
Supplement: Supplementary file 1 — Supplementary Material 1 [file 11571_2025_10252_MOESM1_ESM.docx]

**Supplemental Information**


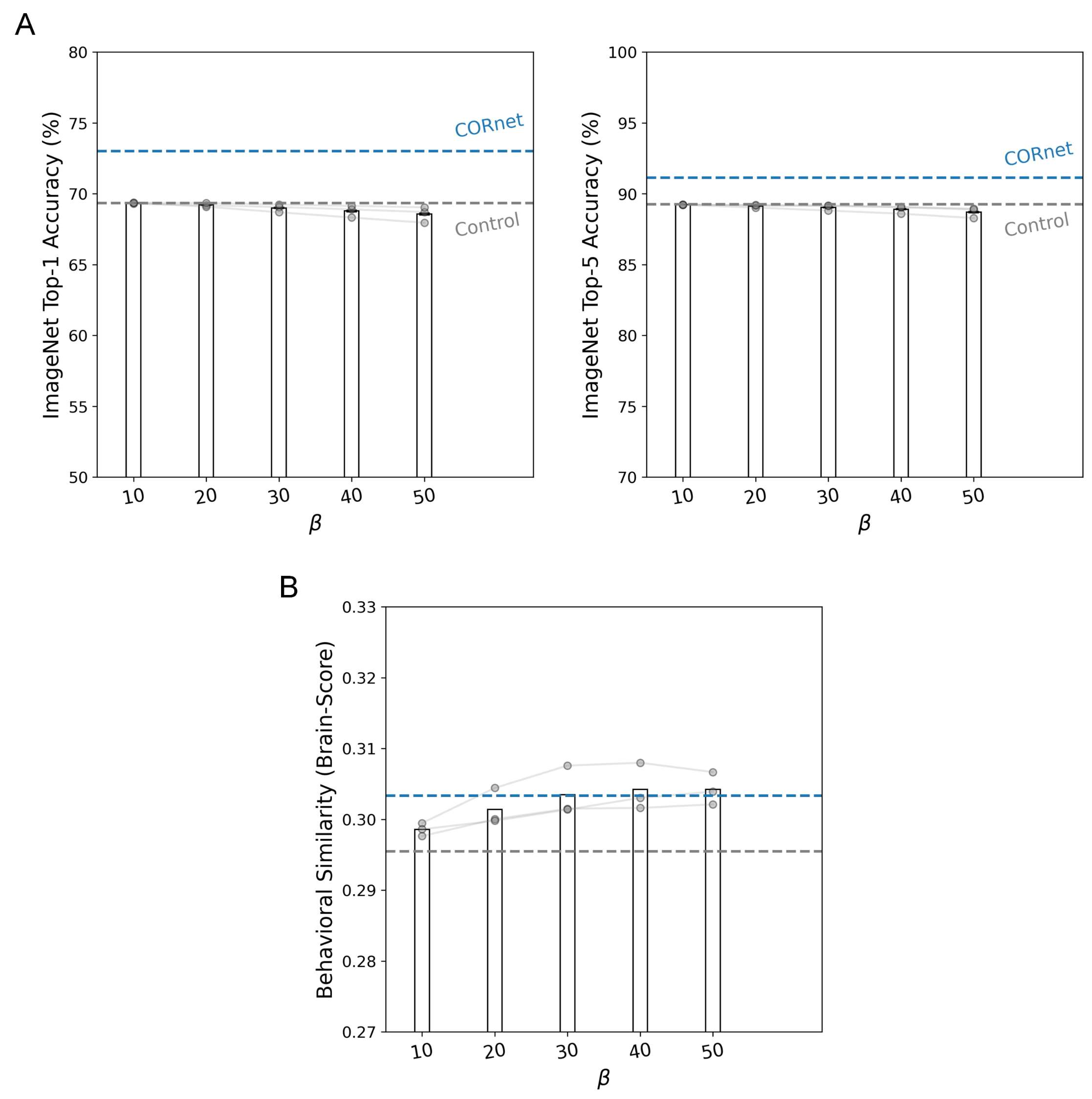


**Figure S1** Classification accuracy on ImageNet and (B) behavior similarity on Brain-Score of ReAlnet-fMRIs at different *β* values.


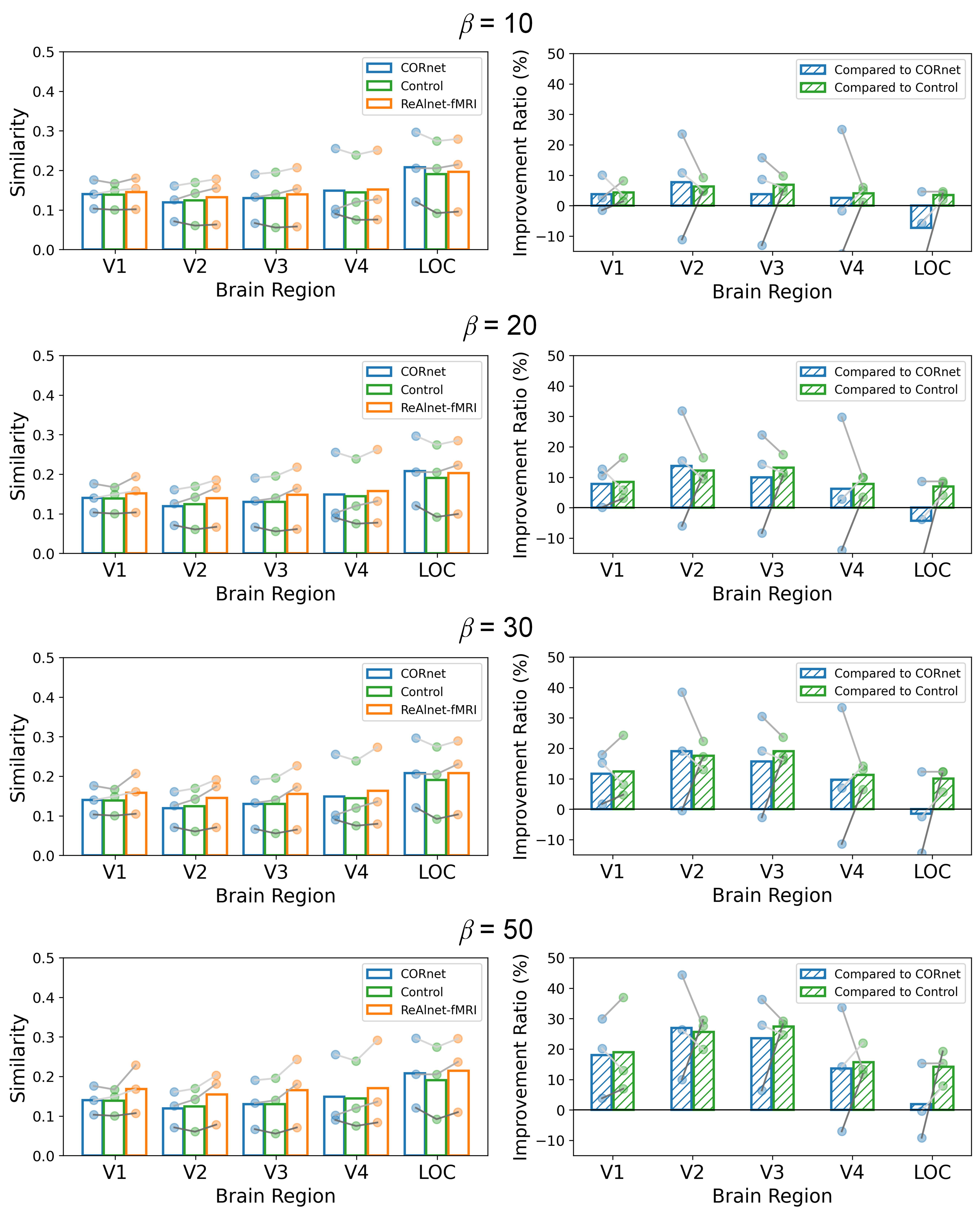


**Figure S2** Within-subject model-fMRI similarity and similarity improvement ratio on natural images of ReAlnet-fMRIs with *β* = 10, 20, 30, and 50. Each circle dot indicates an individual ReAlnet-fMRI.


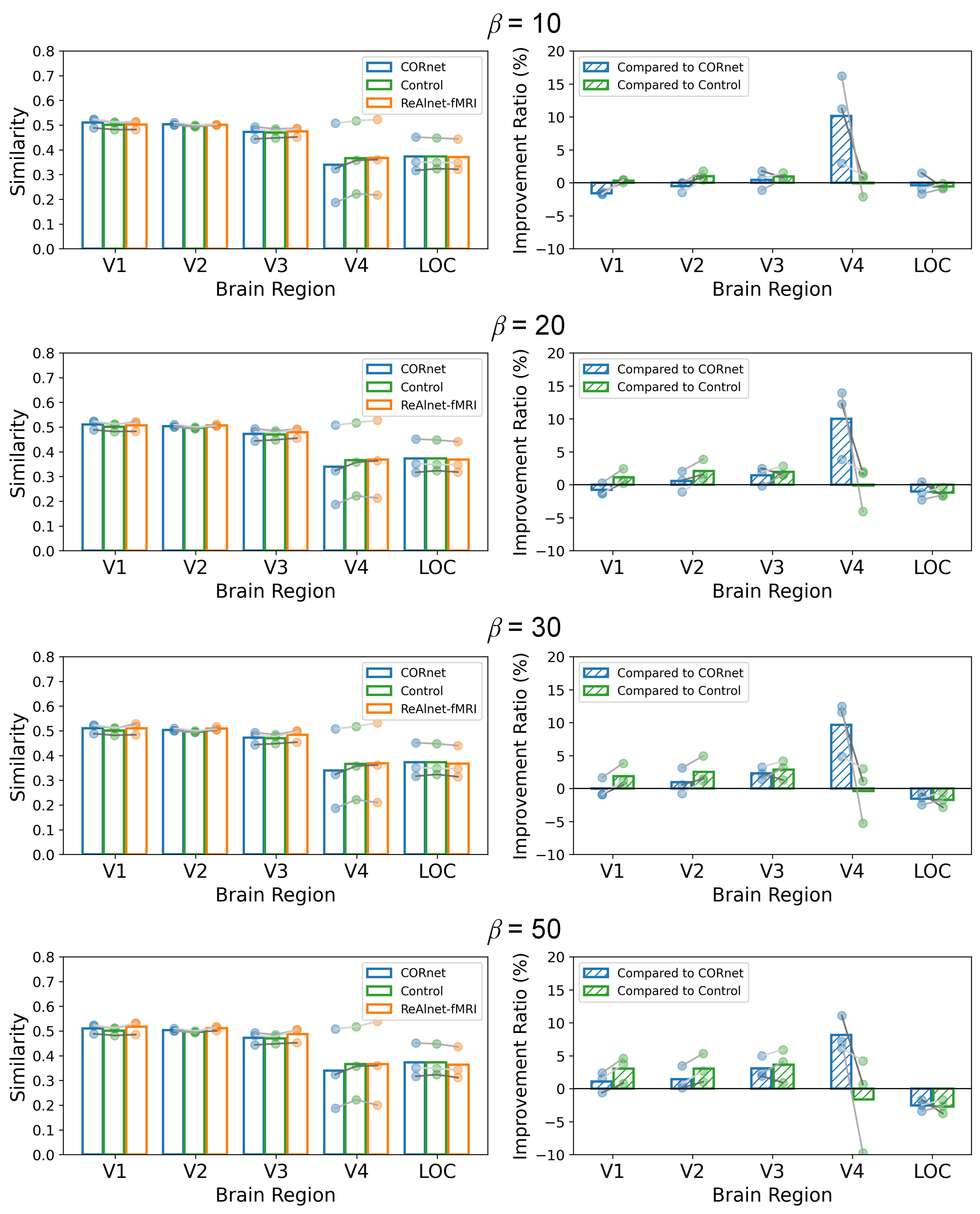


**Figure S3** Within-subject model-fMRI similarity and similarity improvement ratio on artificial shape images of ReAlnet-fMRIs with *β* = 10, 20, 30, and 50. Each circle dot indicates an individual ReAlnet-fMRI.


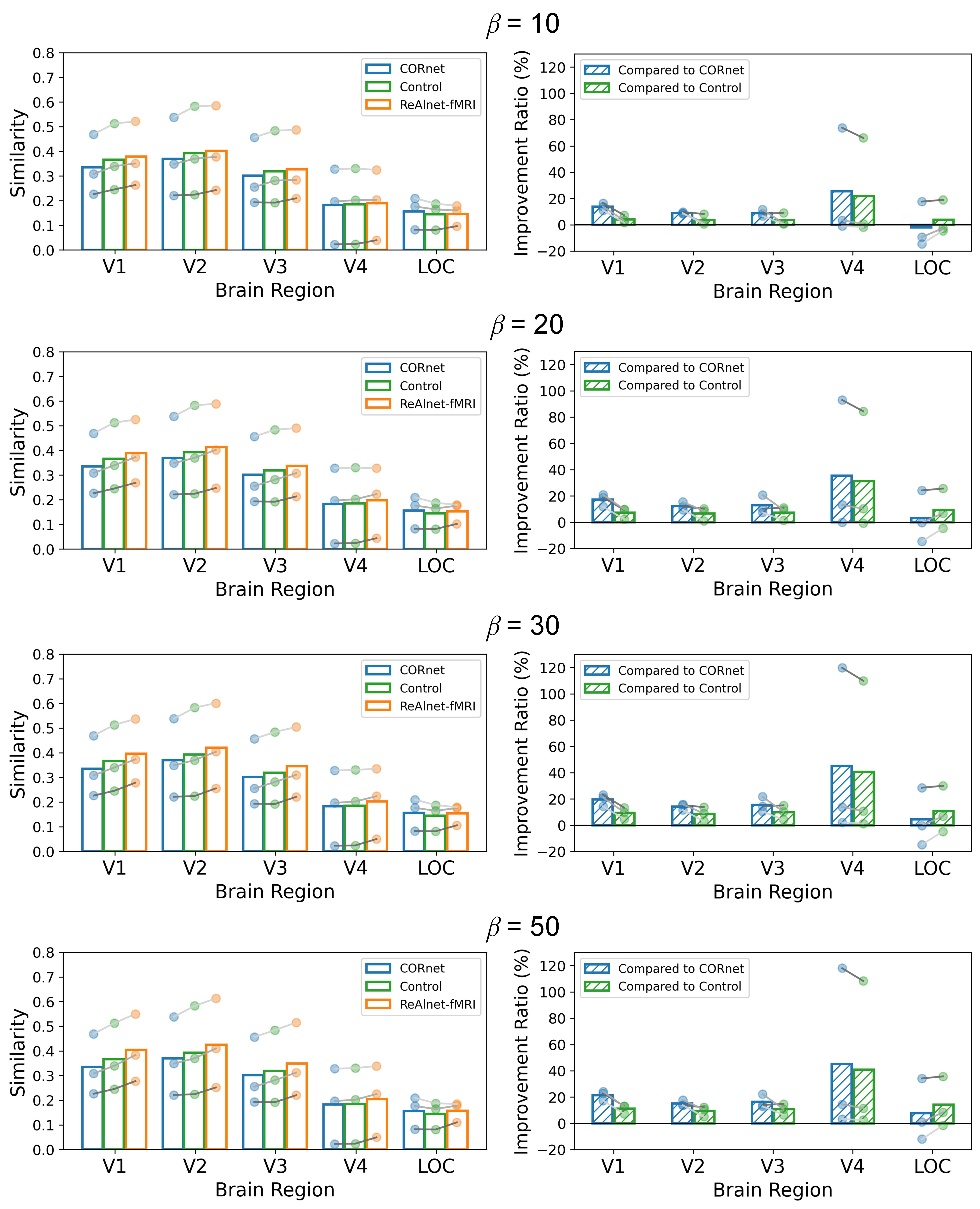


**Figure S4** Within-subject model-fMRI similarity and similarity improvement ratio on alphabetical images of ReAlnet-fMRIs with *β* = 10, 20, 30, and 50. Each circle dot indicates an individual ReAlnet-fMRI.





**Figure S5** Across-subject model-fMRI similarity and similarity improvement ratio of ReAlnet-fMRIs with *β* = 10, 20, 30, and 50. Each circle dot indicates a subject from *Horikawa fMRI dataset*.


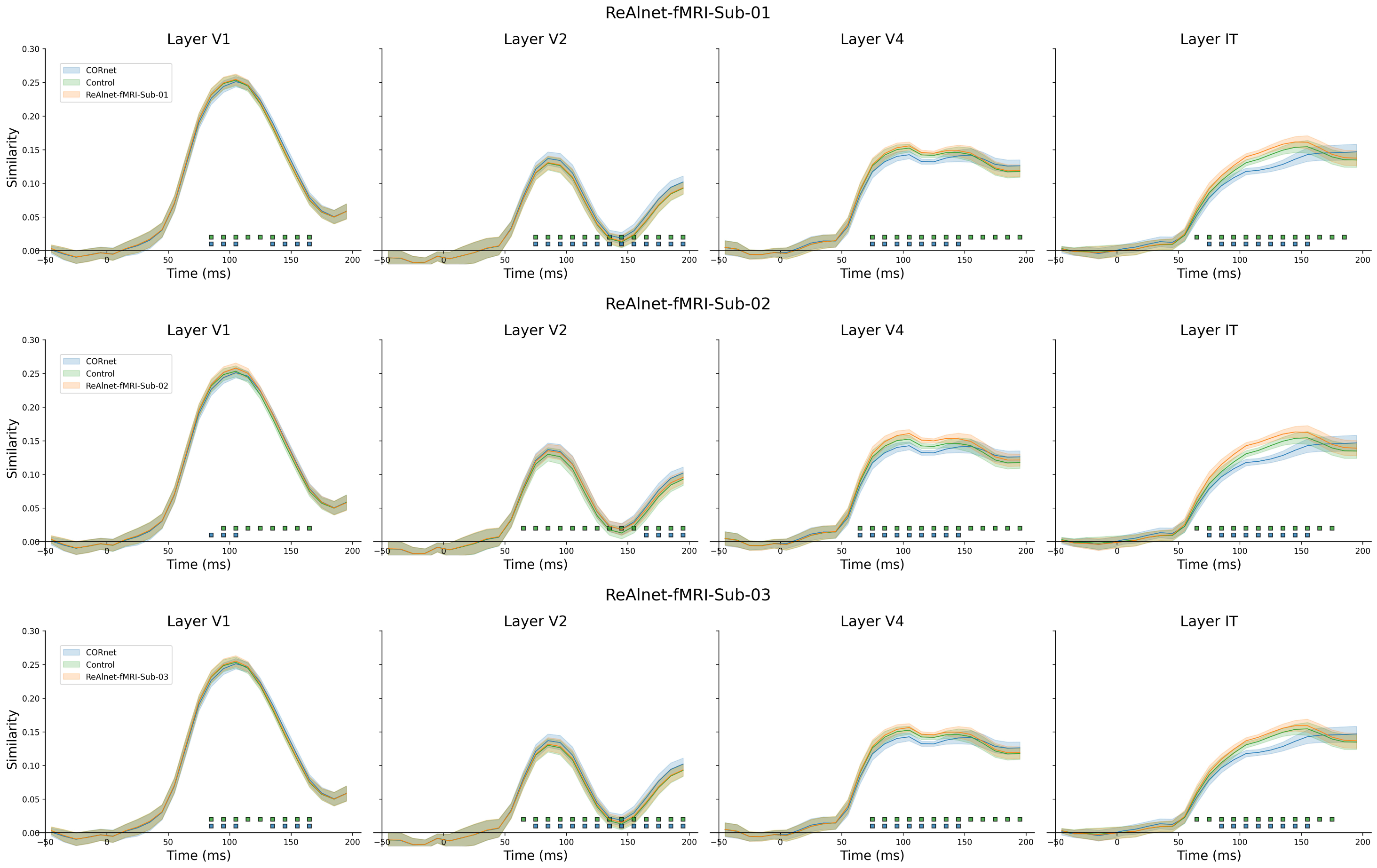


**Figure S6** Across-subject temporal model-EEG similarity of ReAlnet-fMRIs with *β* = 10. Blue and green square dots with black outlines at the bottom indicate the timepoints where ReAlnet-fMRI vs. CORnet and ReAlnet-fMRI vs. Control were significantly different (*p* < .05). Shaded area reflects ± SEM.


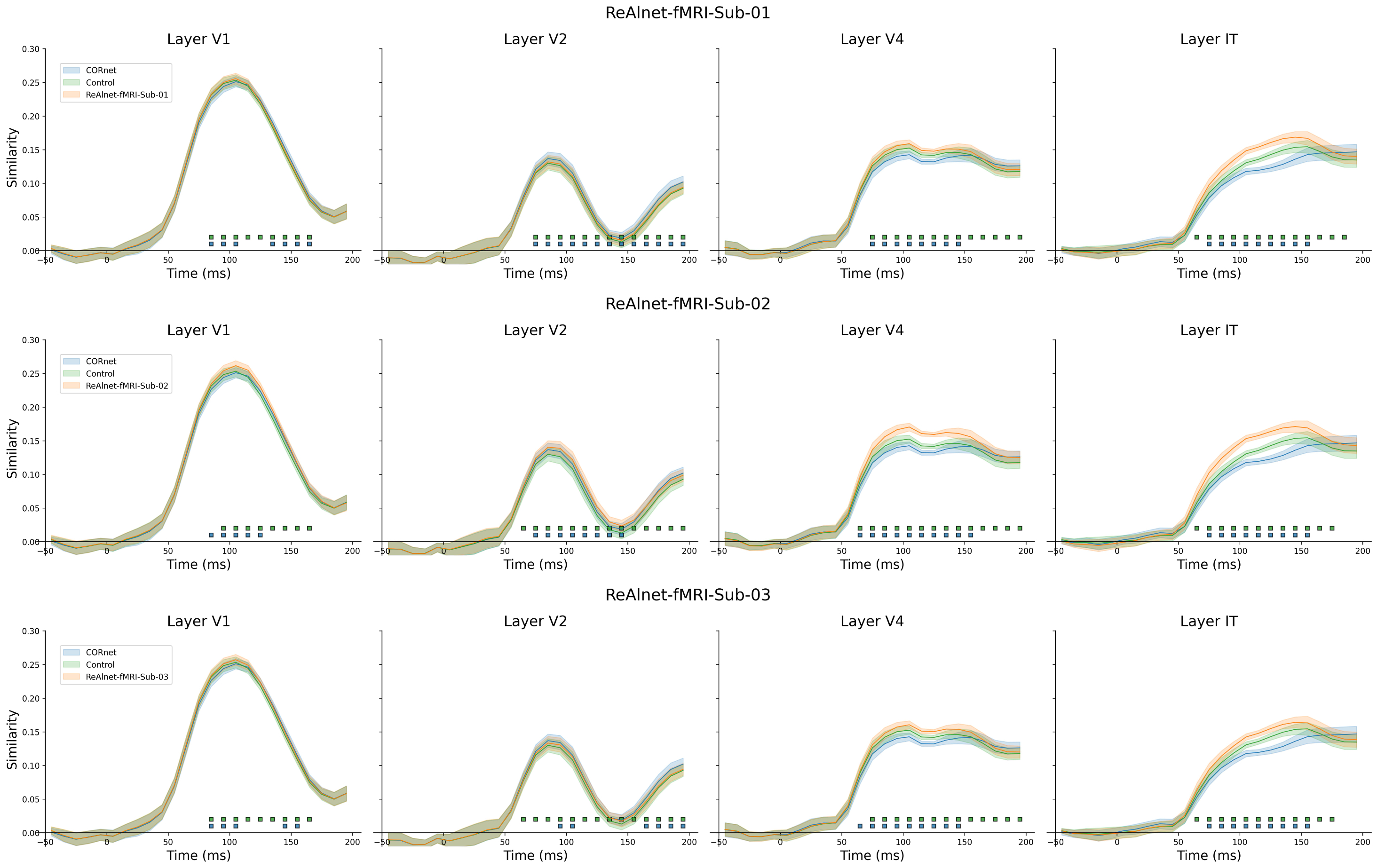


**Figure S7** Across-subject temporal model-EEG similarity of ReAlnet-fMRIs with *β* = 20. Blue and green square dots with black outlines at the bottom indicate the timepoints where ReAlnet-fMRI vs. CORnet and ReAlnet-fMRI vs. Control were significantly different (*p* < .05). Shaded area reflects ± SEM.


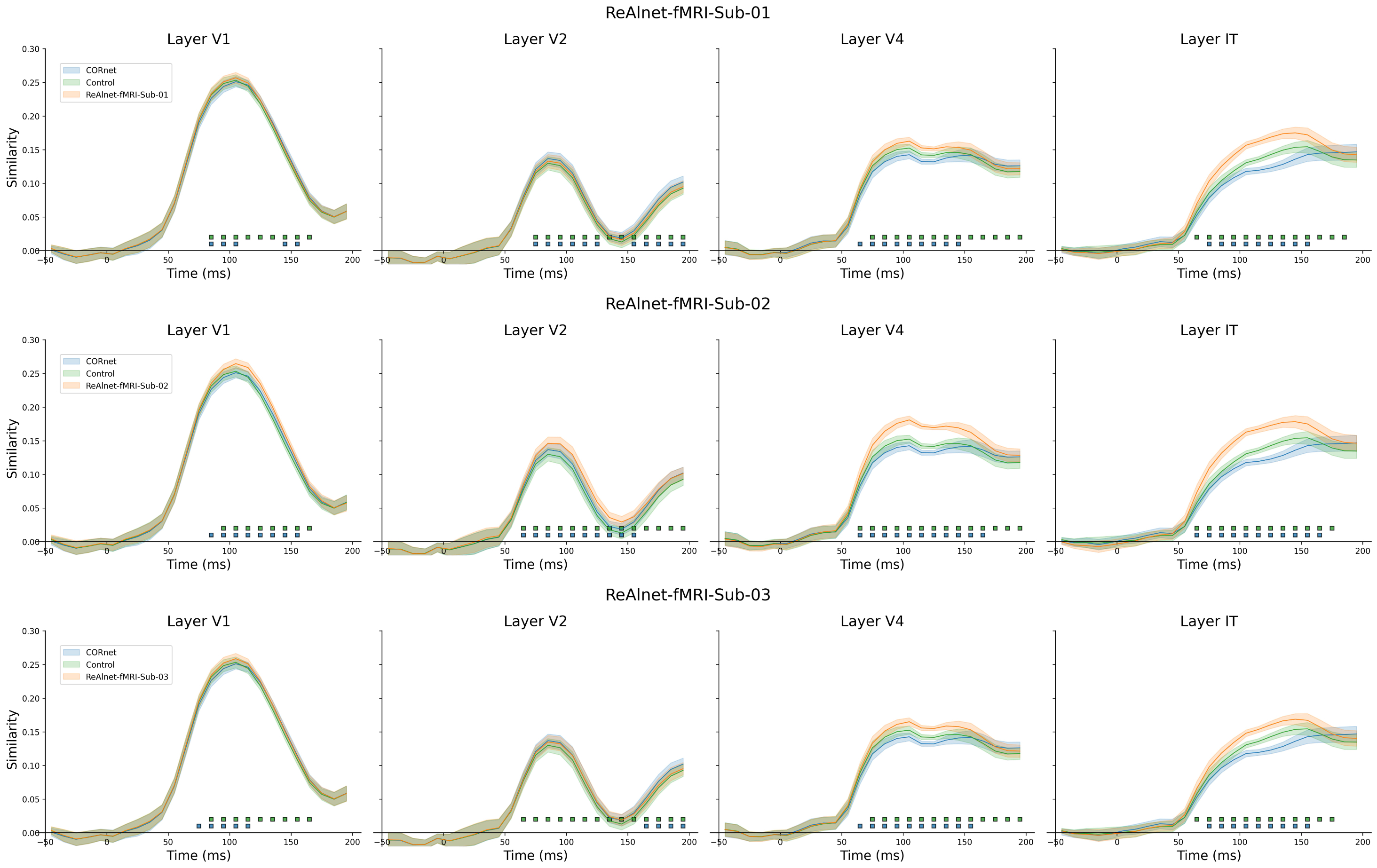


**Figure S8** Across-subject temporal model-EEG similarity of ReAlnet-fMRIs with *β* = 30. Blue and green square dots with black outlines at the bottom indicate the timepoints where ReAlnet-fMRI vs. CORnet and ReAlnet-fMRI vs. Control were significantly different (*p* < .05). Shaded area reflects ± SEM.


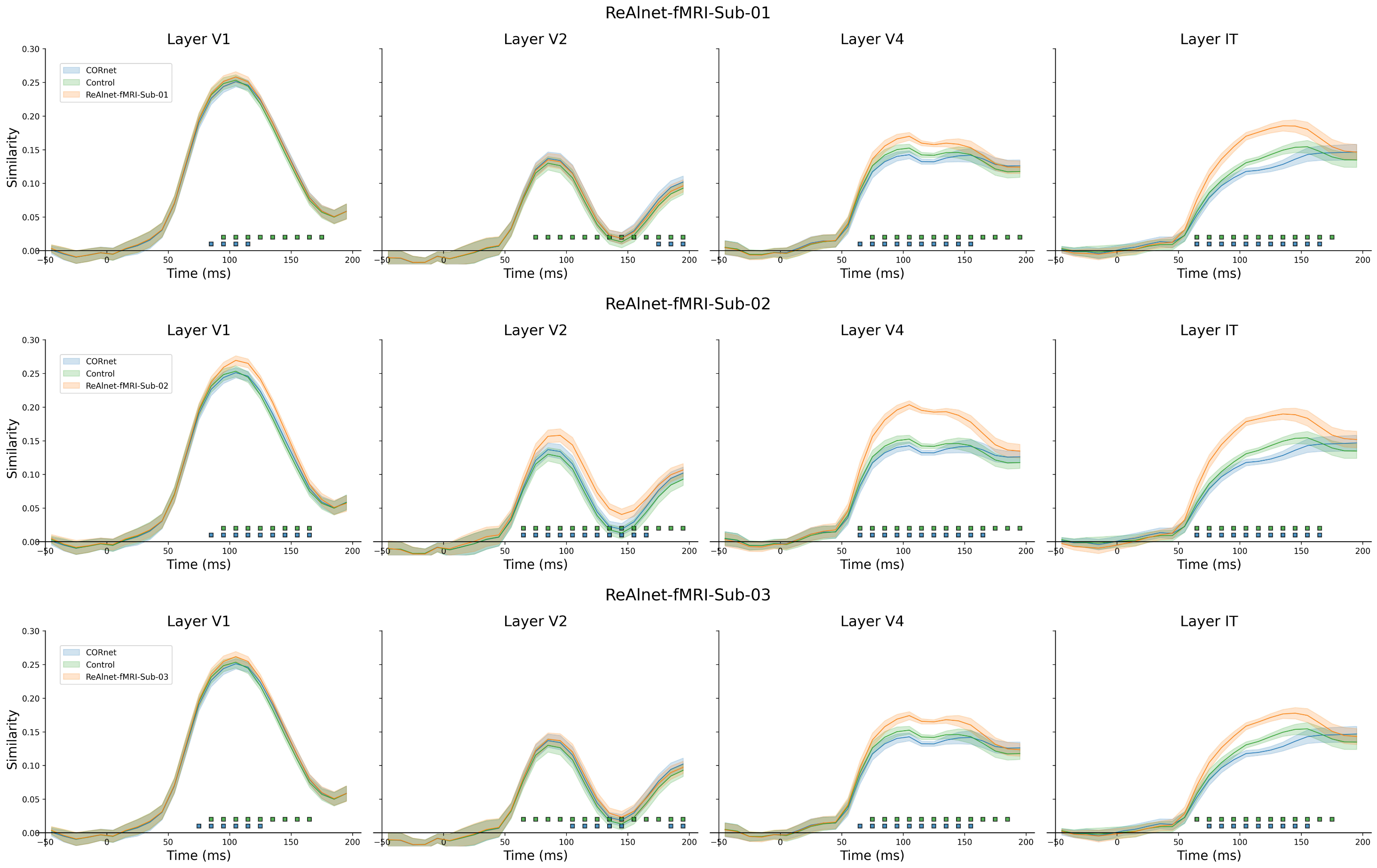


**Figure S9** Across-subject temporal model-EEG similarity of ReAlnet-fMRIs with *β* = 50. Blue and green square dots with black outlines at the bottom indicate the timepoints where ReAlnet-fMRI vs. CORnet and ReAlnet-fMRI vs. Control were significantly different (*p* < .05). Shaded area reflects ± SEM.


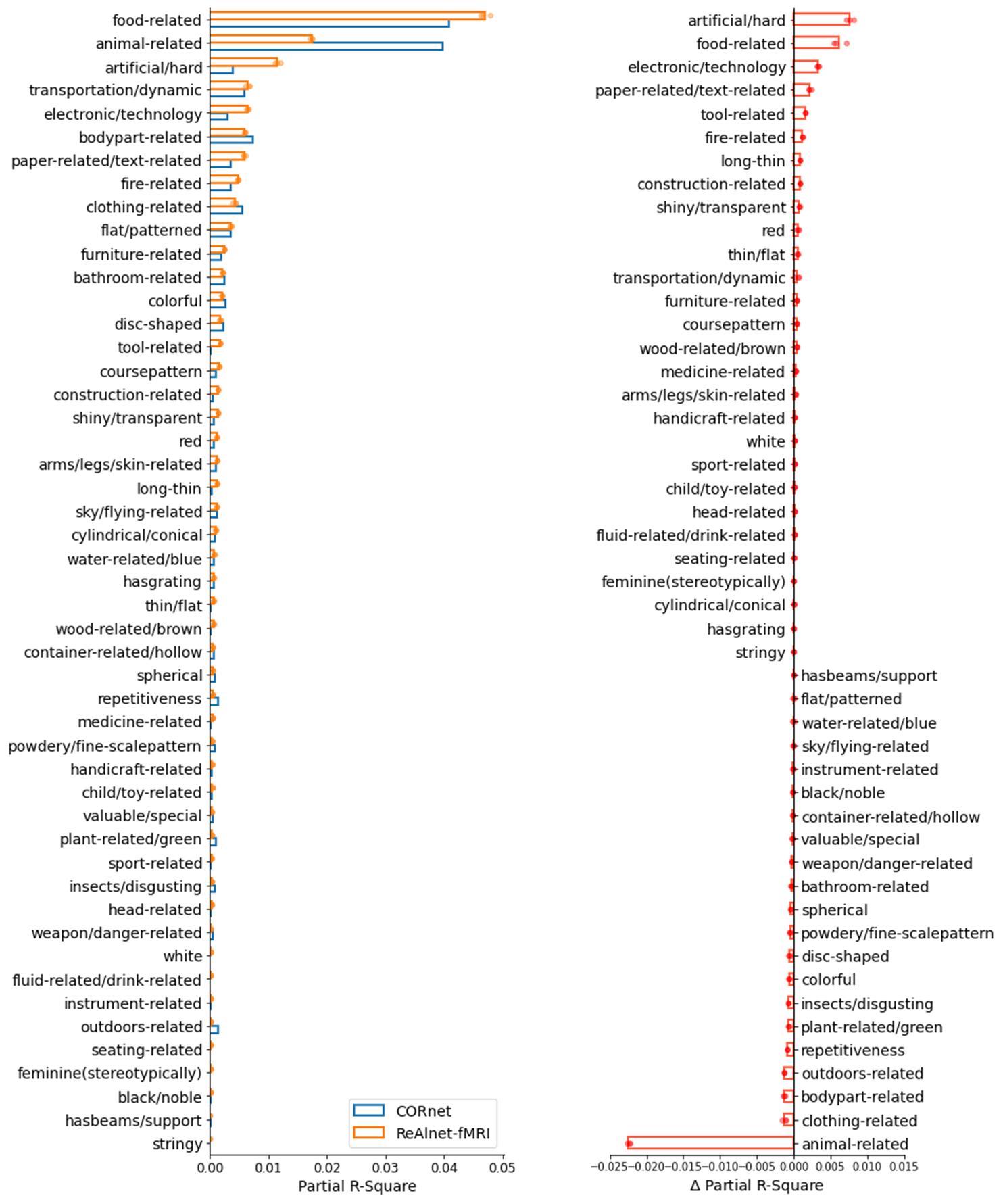


**Figure S10** Internal representations in ReAlnet-fMRIs with *β* = 10 and CORnet. Each circle dot indicates an individual ReAlnet-fMRI.


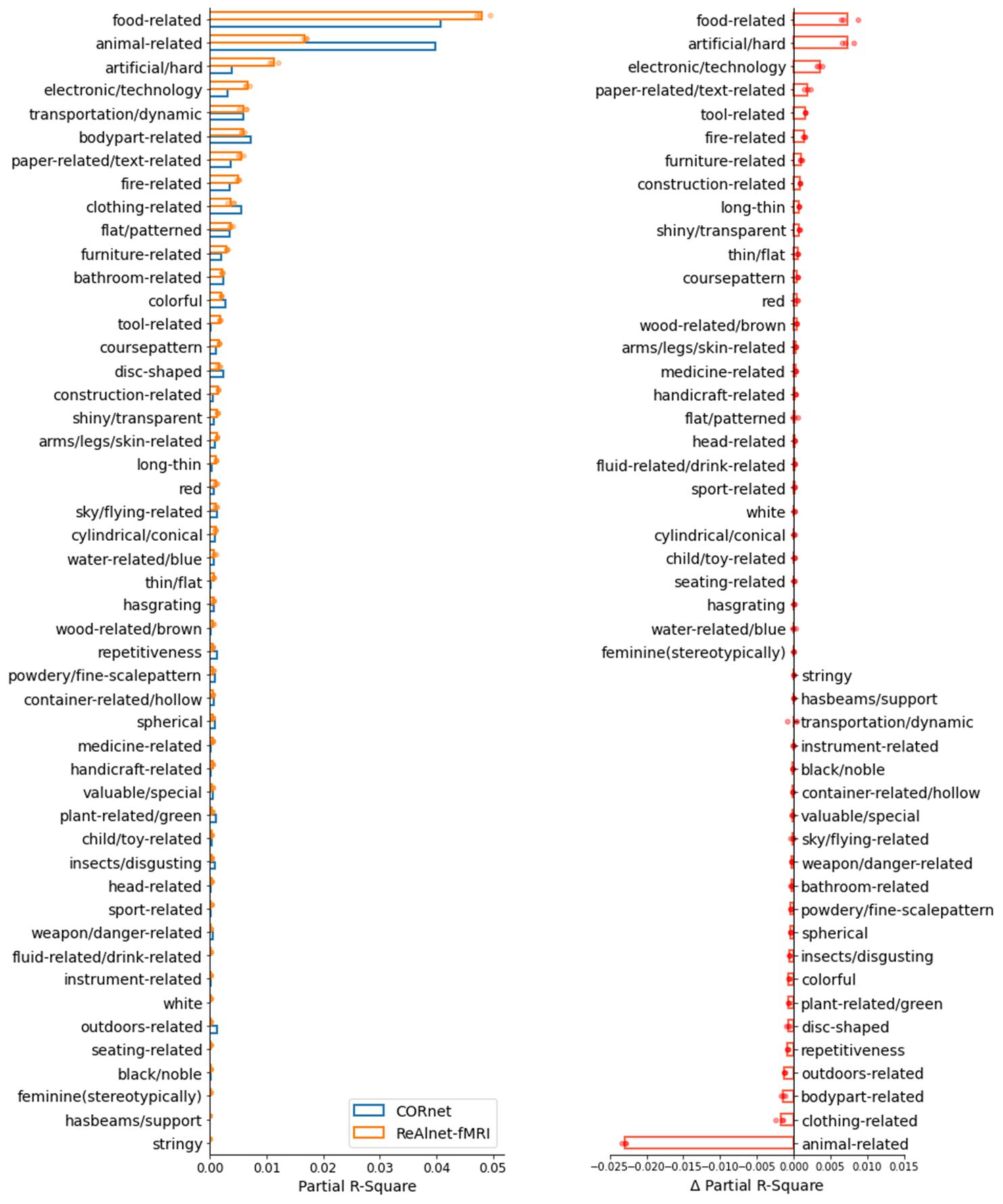


**Figure S11** Internal representations in ReAlnet-fMRIs with *β* = 20 and CORnet. Each circle dot indicates an individual ReAlnet-fMRI.


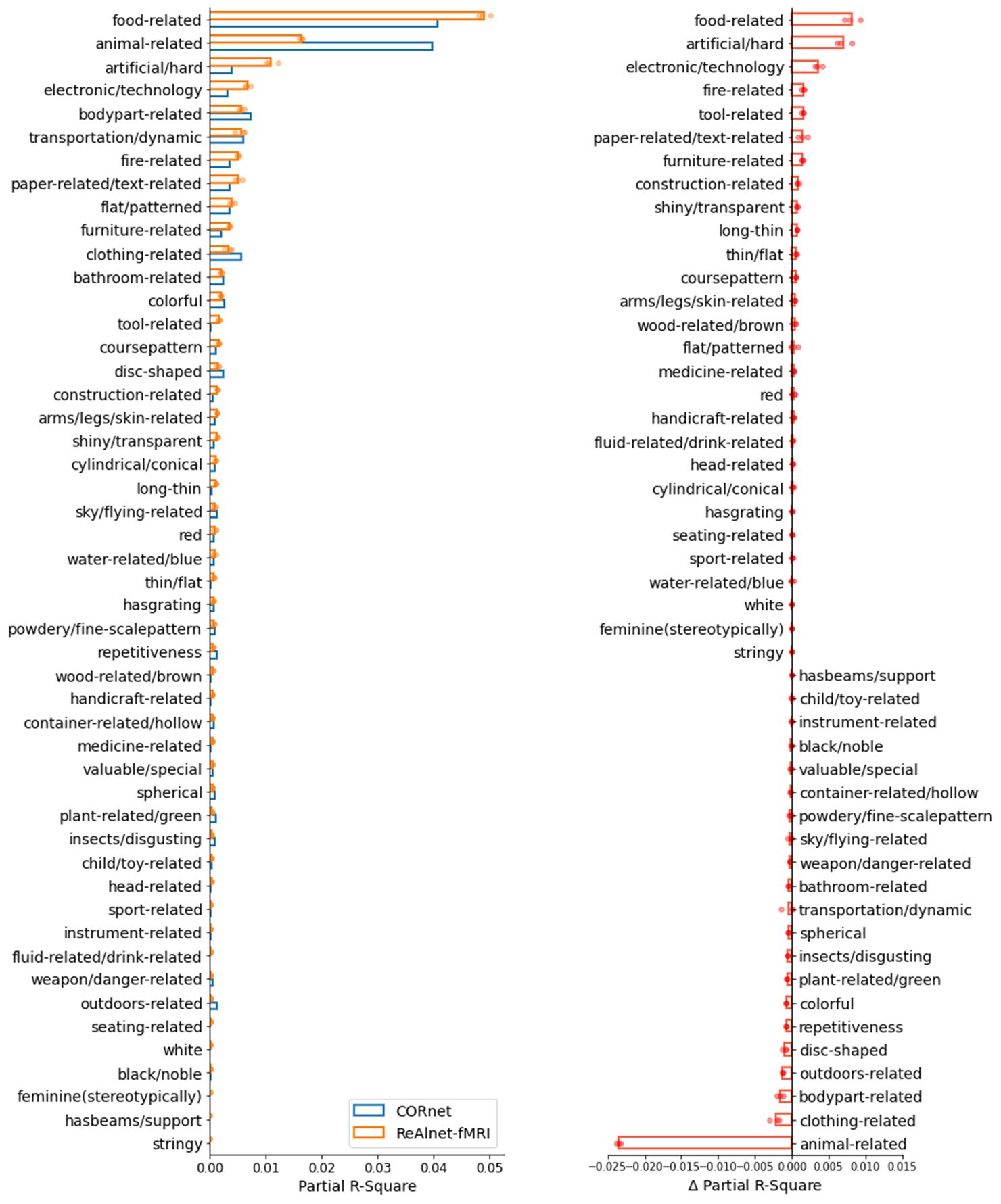


**Figure S12** Internal representations in ReAlnet-fMRIs with *β* = 30 and CORnet. Each circle dot indicates an individual ReAlnet-fMRI.


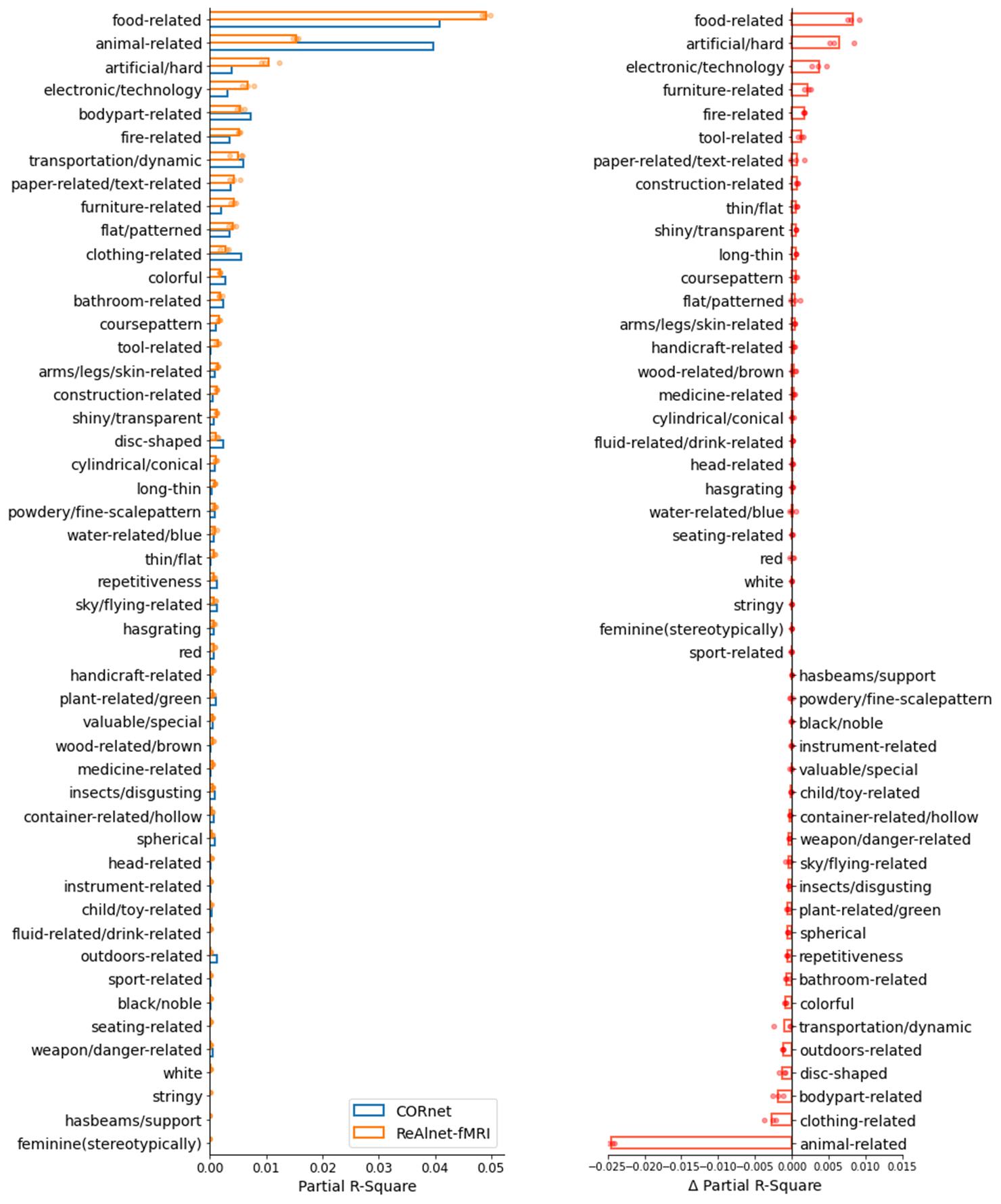


**Figure S13** Internal representations in ReAlnet-fMRIs with *β* = 50 and CORnet. Each circle dot indicates an individual ReAlnet-fMRI.





**Figure S14** Enhanced representations in ReAlnet-fMRIs with *β* = 40 compared to CORnet across Layer V1, V2, and V4. Each circle dot indicates an individual ReAlnet-fMRI.
